# Consistent changes in muscle metabolism underlie dive performance across multiple lineages of diving ducks

**DOI:** 10.1098/rspb.2023.1466

**Published:** 2023-09-27

**Authors:** Elizabeth R. Schell, Kevin G. McCracken, Graham R. Scott, Jeff White, Philip Lavretsky, Neal J. Dawson

**Affiliations:** ^1^ Department of Biology, University of Miami, Coral Gables, FL 33146, USA; ^2^ Department of Marine Biology and Ecology, Rosenstiel School of Marine, Atmospheric, and Earth Science, University of Miami, Miami, FL 33149, USA; ^3^ Human Genetics and Genomics, University of Miami Miller School of Medicine, Miami, FL 33136, USA; ^4^ University of Alaska Museum, University of Alaska Fairbanks, Fairbanks, AK 99775, USA; ^5^ Department of Biology, McMaster University, Hamilton, Ontario, Canada L8S 4K1; ^6^ Department of Biological Sciences, University of Texas El Paso, El Paso, TX 79968, USA; ^7^ School of Biodiversity, One Health & Veterinary Medicine, University of Glasgow, Glasgow, G12 8QQ, UK

**Keywords:** aerobic metabolism, breath-hold diving, mitochondrial energetics, oxidative phosphorylation, lipid oxidation, electron transport system

## Abstract

Diving animals must sustain high activity with limited O_2_ stores to successfully capture prey. Studies suggest that increasing body O_2_ stores supports breath-hold diving, but less is known about metabolic specializations that underlie underwater locomotion. We measured maximal activities of 10 key enzymes in locomotory muscles (gastrocnemius and pectoralis) to identify biochemical changes associated with diving in pathways of oxidative and substrate-level phosphorylation and compared them across three groups of ducks—the longest diving sea ducks (eight spp.), the mid-tier diving pochards (three spp.) and the non-diving dabblers (five spp.). Relative to dabblers, both diving groups had increased activities of succinate dehydrogenase and cytochrome c oxidase, and sea ducks further showed increases in citrate synthase (CS) and hydroxyacyl-CoA dehydrogenase (HOAD). Both diving groups had relative decreases in capacity for anaerobic metabolism (lower ratio of lactate dehydrogenase to CS), with sea ducks also showing a greater capacity for oxidative phosphorylation and lipid oxidation (lower ratio of pyruvate kinase to CS, higher ratio of HOAD to hexokinase). These data suggest that the locomotory muscles of diving ducks are specialized for sustaining high rates of aerobic metabolism, emphasizing the importance of body O_2_ stores for dive performance in these species.

## 1. Introduction

Breath-hold diving presents air-breathing organisms with a suite of physiological challenges, including tissue hypoxemia due to apnea during the dive, recovery after the dive and potential amplification of heat loss in cool water. Supporting the high levels of aerobic metabolism necessary for successful foraging under these conditions is physiologically taxing due to the decreasing availability of oxygen (O_2_) reserves as dive time progresses, creating a potential mismatch between tissue O_2_ supply and demand [1].

The magnitude of the potential mismatch between O_2_ supply and demand has a strong bearing on dive capacity. Aerobic metabolism yields far more ATP per mole of fuel than anaerobic metabolism, and previous studies have shown that most foraging dives by seals and penguins stay within this aerobic threshold [2–4]. The aerobic dive limit is extended in many diving animals by virtue of having increased body O_2_ stores, often achieved via increases in blood haemoglobin (Hb) content and muscle myoglobin (Mb) content [5–8]. It is also possible that reductions in metabolic O_2_ demands could offset the potential mismatch between O_2_ supply and demand during diving, thus extending dive times. However, previous studies have shown that activities of aerobic enzymes in locomotory muscles, specifically citrate synthase (CS) and hydroxyacyl-CoA dehydrogenase (HOAD), are often increased relative to non-locomotory muscles among diving species, as well as at the advent of diving (e.g. juvenile penguins going to sea for the first time) [9–11]. This suggests that at least for skeletal muscles, high capacities for aerobic metabolism are needed to synthesize adequate amounts of ATP to fuel diving locomotion and/or post-dive recovery.

Diving ducks represent a compelling and taxonomically diverse system in which to study diving adaptations. The diving ducks are a polyphyletic group in the largest waterfowl sub-family *Anatinae*, and include the sea ducks (tribe *Mergini*) and the pochards (tribe *Aythyini*) [12]. Recent phylogenetic studies based on complete mitochondrial genomes suggested that the sea ducks represent the more basal of these two lineages, branching from the rest of the *Anatinae* approximately 18−20 Ma, while the pochards branched approximately 15−16 Ma within the *Anatinae* [13,14]. Diving thus evolved separately in the sea duck and pochard clades, allowing us to investigate whether they have converged on similar diving phenotypes as seen in other aspects of their morphology [15]. Sea ducks also contain many of the species considered to be the strongest divers in terms of both time and depth, including the long-tailed duck (*Clangula hyemalis*), which has been caught in gill nets as deep as 55 m [16]. Across the sea ducks and pochards represented here, anecdotal observations suggest that both dive time and maximum depth vary widely, although there has been no standardized methodology for accurately measuring these variables for most species. The large variation in dive time across closely related species allows us to investigate whether diving phenotypes vary by species with progressively longer dive times.

However, when conducting interspecies comparisons, there is the potential for phenotypic flexibility among individuals to confound results. Traits such as enzyme activity can vary throughout the annual cycle of waterfowl in response to activities like migration [17], although consistent biochemical patterns have yet to be identified across birds [18]. Failure to account for this flexibility can therefore influence interspecific comparisons. One way to minimize this is to standardize sample collection (e.g. using all wild-caught individuals, sampling during one point in the annual cycle) [19]. While phenotypic flexibility among individuals is unavoidable, this strategy reduces its potential effects when conducting cross-species comparisons.

Here, we investigate the diving phenotype of multiple species by examining how the evolution of diving has restructured pathways of aerobic metabolism and fuel use in the locomotory muscle. We compared the maximal activities of 10 key enzymes across pathways of glycolysis, fatty acid oxidation, the tricarboxylic acid (TCA) cycle and oxidative phosphorylation in 16 species of ducks with variable dive capabilities: the longest and deepest diving sea ducks (eight spp.), the mid-tier diving pochards (three spp.) and the non-diving dabblers (five spp.). The 11 diving species studied here are cited primarily as leg-propelled divers, although recent evidence shows that the common merganser (*Mergus merganser*) also uses wing propulsion [20], so all measurements were taken in both the gastrocnemius and the pectoralis muscles. Owing to the interrelatedness and shared evolutionary history of these species, all statistical comparisons were completed using a phylogenetic framework [21,22].

## 2. Methods

### (a) Sample overview

**Figure 1.**
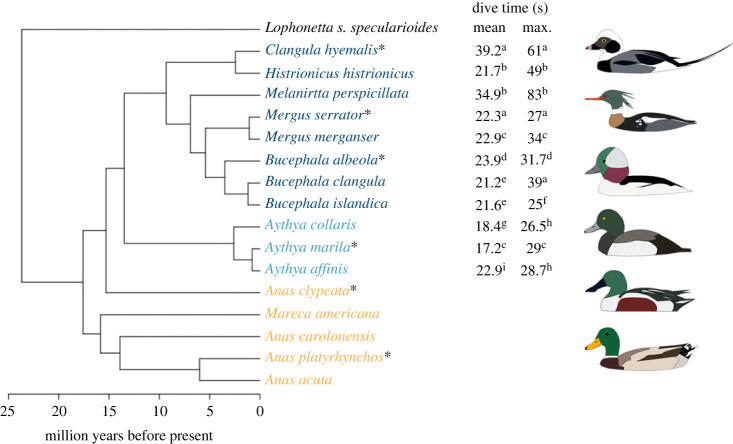
Phylogeny for the 16 species studied here, generated using 10 731 overlapping autosomal ddRAD-seq loci in BEAST v. 2.5.2. Branch lengths were assigned using treePL. Asterisks indicate corresponding species graphic. Tribes are indicated by colour: sea ducks (dark blue), pochards (teal), dabblers (gold). Dive times were obtained from several sources in the literature, represented by the following superscripts: a, [23]; b, [24]; c, [25]; d, (Boone JL, LaRue EA 1998, unpublished data); e, [26]; f, [27]; g, [28]; h, [29]; i, [30].

Tissue samples were taken from up to 10 individuals across the 16 species of diving and non-diving ducks (figure 1). All birds were sampled in Alaska, USA, from ponds and rivers around Fairbanks, the Denali Highway and the Dalton Highway, as well as Prince William Sound and Kodiak across a 3-year period from 2019 to 2021 (electronic supplementary material, table S1). We collected samples during spring migration, which lasts from early March to late May [31], collecting most species in May and early June, except for harlequin ducks (*Histrionicus histrionicus*) and surf scoters (*Melanitta perspicillata*), which were collected in March. We collected species during the same point in their annual cycle to limit the potential influence of phenotypic plasticity on our interspecific analyses. Tissue samples from the pectoralis and gastrocnemius muscles were immediately dissected from an intermediate depth (50% muscle depth) and flash frozen in liquid N_2_. Samples were then stored at −80°C until enzymatic analysis was completed (see below).

All specimen collection and import/export were conducted with permits from the U.S. Fish and Wildlife Service (MB33283C, MB812229, MB836720), Alaska Department of Fish and Game (19–091, 19–153, 20–033, 20–091, 21–103), Scottish Animal Health and Welfare Division (TARP(S) 2022/04), and Institutional Animal Care and Use Committee (IACUC) from the University of Miami (17–107, 20–090).

### (b) Species phylogeny

We reconstructed a phylogenetic species tree by downloading and aligning double digest restriction-site associated DNA sequencing (ddRADseq) data [32] for the 16 study species and an outgroup species. The ddRADseq data were first aligned using bioinformatics pipelines outlined in Lavretsky *et al*. [33] using Python scripts to automate sequence filtering, alignment and genotyping using a combination of Trimmomatic [34], Burrows Wheeler Aligner v. 07.15 [35] and Samtools v. 1.7 [34]. Using VCFtools v. 0.1.15 [36], we further filtered the sequences for missing data (greater than 20% of samples), minimum base-pair depth of coverage of 5× (10× per genotype) and per base PHRED quality scores of greater than or equal to 30. All sequences were aligned to a chromosomally assembled wild mallard (*Anas platyrhychos*) genome [37], which allowed us to distinguish between autosomal and sex-linked ddRAD-seq loci.

To reconstruct a species tree, we employed the SNAPP function [38] in the program *BEAST v. 2.5.2 [39]. In short, SNAPP uses bi-allelic single nucleotide polymorphisms (SNPs) to derive a posterior distribution of putative species trees by estimating the probability of allele frequency changes across nodes given the data. To do so, we first filtered our ddRAD-seq dataset for bi-allelic SNPs using PLINK v. 1.9 [40], as well as for singletons (i.e. minimum allele frequency (--maf 0.018), any SNP missing greater than or equal to 20% of data across samples (--geno 0.2), and any SNPs found to be in linkage disequilibrium (LD) (--indep-pairwise 2 1 0.5). We employed the Hasegawa–Kishino–Yano (HKY) substitution model [41] with a gamma distribution across sites, and with five of these having some proportion of invariable sites [32]. We employed a strict molecular clock. SNAPP analysis was run for 100 000 000 iterations, with a burn-in of 100 000 steps, and sampling occurring every 1000 iterations, to ensure that the effective sample sizes (ESS) across parameters were greater than or equal to 100. Burn-in was set to 10% of the total number of sampled trees, and the final species tree was constructed in R using phytools [22]. Finally, we dated the phylogeny using treePL [42], with divergence times obtained from TimeTree [43].

### (c) Enzymatic assays

**Figure 2.**
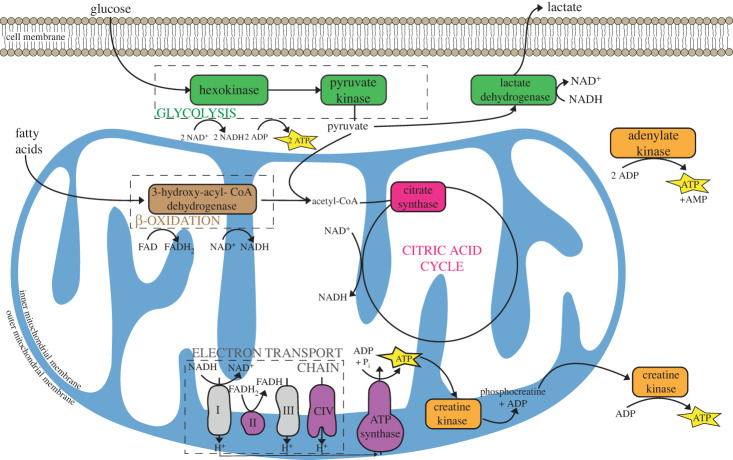
Schematic of the metabolic pathways investigated here. The 10 enzymes assayed are highlighted in green (glycolysis and lactate production), brown (beta-oxidation), pink (citric acid cycle), purple (electron transport system; succinate dehydrogenase is complex II; cytochrome c oxidase is complex IV), and orange (substrate-level phosphorylation).

The maximal activities of the 10 metabolic enzymes shown in figure 2 were assayed as previously described [44,45]. These included the first and last steps of glycolysis (hexokinase [HK] and pyruvate kinase [PK]); lactate dehydrogenase (LDH), which catalyses the interconversion of pyruvate to lactate; citrate synthase (CS), an enzyme in the TCA cycle and a common marker for mitochondrial volume density; succinate dehydrogenase (SDH), an enzyme in the TCA cycle and Complex II of the electron transport system; cytochrome c oxidase (COX), Complex IV and the terminal O_2_ consumer in the electron transport system, and a common marker of mitochondrial cristae density; ATP synthase (ATPSyn), Complex V of oxidative phosphorylation; hydroxyacyl-CoA dehydrogenase (HOAD), which catalyses an important step in fatty acid oxidation; and adenylate kinase (AK) and creatine kinase (CK), both involved in substrate-level phosphorylation and intracellular transfer of high-energy phosphates.

Briefly, frozen tissue samples were homogenized in 20 volumes of ice-cold homogenization buffer (100 mM potassium phosphate, 1 mM EGTA, 1 mM EDTA, 0.1% Triton X-100; pH 7.2), then centrifuged for 2 min at 2000 r.p.m. The supernatant was collected and used in the subsequent assays. Each assay was conducted at avian body temperature (41°C) with substrate concentrations previously found to be saturating [44]. The substrates and their concentrations used for each enzymatic assay are listed in electronic supplementary material, table S2. All chemicals were sourced from Merck (Darmstadt, Germany) unless otherwise noted. Assays were run in triplicate, and the change in absorbance was measured using a Spectramax Plus 384 spectrophotometer (Molecular Devices, Sunnyvale, CA, USA). The maximal activity for each enzyme was calculated as the difference between the reaction rate with all substrates present minus the background reaction rate (the rate in the presence of an inhibitor or without the key substrate) and is reported as units of micromole substrate per gram tissue (U g^−1^) per minute.

### (d) Haemoglobin and myoglobin concentration

Haemoglobin and myoglobin concentrations are reported in Schell *et al*. [46]. Upon collection, whole blood samples were immediately extracted from birds via cardiac puncture. Blood Hb concentration ([Hb]) was then analysed using a Hemocue 201 + Analyzer (HemoCue America, Brea, CA, USA), which measures absorbance at 570 and 880 nm, and corrected using the avian correction factor of −1 g dl^−1^ [47,48]. Packed cell volume, or haematocrit (Hct), was measured as the average of four 75 mm heparinized microcentrifuge tubes capped and spun for 5 min in a ZIPocrit centrifuge (LW Scientific, Lawrenceville, GA, USA).

Myoglobin concentration was determined using a modified Reynafarje method [49]. Frozen tissue samples were homogenized in 19.5 volumes of ice-cold homogenization buffer (40 mM potassium phosphate; pH 6.6), then centrifuged for 99 min at 13 700 r.p.m. and 4°C [44]. The resulting supernatant was transferred to a 25 ml boiling flask and exposed to carbon monoxide (CO) for 8 min under constant rotation. After an addition of sodium dithionite, CO was bubbled in for a further 2 min to ensure complete reduction of the myoglobin. Samples were then diluted 19.5× with homogenization buffer, and the optical density of each sample was measured in triplicate with a blank at 538 and 568 nm using a VWR V1200 Spectrophotometer (VWR, Radnor, PA, USA). Myoglobin concentration ([Mb]; mg g^−1^) was then determined based on the following equation: [Mb] = (OD_538_ − OD_568_) × 112.6 [49].

### (e) Dive times

A comprehensive literature search for dive times was performed for each species. When multiple reported times were found, the time chosen was prioritized by accounts reporting wild birds diving in their natural habitat over studies of captive birds. Where possible, data from the same study were used for multiple species to account for observer bias. Dive times are reported as mean dive time and maximum dive time, and we used mean dive time for each species in our linear mixed models. Dabblers were assigned dive times of 0 s (figure 1).

### (f) Analysing physiological changes in a phylogenetic context

All analyses were conducted to account for phylogenetic non-independence [50] using the R Statistical Software (v. 4.2.1) [51]. Because closely related species are not statistically independent, we grouped species into the three clades (i.e. tribes) with replicates within each clade (sea ducks, pochards and dabblers). We first identified which enzymes showed phylogenetic signal by testing for Pagel's *λ* [52]. Principal components analysis (PCA) was next used to visualize and describe differences between tribes and species across all enzymes in the gastrocnemius only. Phylogenetic ANOVA (phytools, nsim = 1000) [22] followed by *post hoc* Bonferroni tests were used to make comparisons between sea ducks, pochards and dabblers for all enzymes and ratios of activity between different enzymes. Enzyme ratios were chosen to assess the relative reliance on certain metabolic pathways (e.g. LDH:CS to compare anaerobic versus aerobic metabolism [53], ATPSyn:CS to indicate ATP production relative to total oxidative capacity) [54]. Enzymes identified to be significantly different (*p* < 0.05) or nearly significant (*p* < 0.10) between groups in either the pectoralis or gastrocnemius were used as explanatory variables against dive time in phylogenetic least squares regressions (phytools, correlation = Brownian Motion). Body mass, blood [Hb] and muscle [Mb] were also included because certain enzymes and overall dive time have been shown to scale with these or other variables [55].

## 3. Results

### (a) Species tree phylogeny

Our SNAPP species tree was reconstructed using a total of 10 731 overlapping autosomal ddRAD-seq loci that met our filtering criteria. First, apart from *Anas*, all genera are monophyletic, with some sister species like lesser scaup (*Aythya affinis*) and greater scaup (*Aythya marila*) diverging less than 1 Ma. One difference from other waterfowl phylogenies is the placement of sea ducks and pochards sister to each other, diverging 13.5 Ma, instead of the sea ducks representing the more basal split and pochards being sister to dabblers, which is most likely due to the limited number of species included in our tree. Divergence times of 17.5 Ma for sea ducks correlate well with other phylogenies [14]. Within the sea ducks, all species relationships correlate closely with those found by Lavretsky *et al*. [32], except the long-tail duck and harlequin duck, which our tree shows as sister to each other and the earliest diverging of the sea ducks included here (9.3 Ma). Finally, our phylogeny puts the northern shoveler (*Anas clypeata*) in a clade with the diving pochards and sea ducks over dabblers, which could be due to the smaller number of species included, as northern shoveler is part of the dabbler clade in other trees with wider taxonomic sampling [14,56]. Preliminary analyses determined that our phylogeny did not yield different results from a phylogeny constrained to previously published topologies [13,14].

### (b) Phylogenetic signal

Pagel's *λ* has been found to perform better than Blomberg's *K* under the assumption of the Brownian motion model of trait evolution, so it was chosen to test for phylogenetic signal in all enzymes measured here [57]. Pagel's *λ* indicated that four enzymes showed phylogenetic signal (*λ* significantly different from 0). These included ATPSyn, CS and HOAD in the gastrocnemius (*p*s = 0.03, 0.01 and 0.02, respectively) and SDH in the pectoralis (*p* < 0.001).

### (c) Enzyme activities in the gastrocnemius

**Figure 3.**
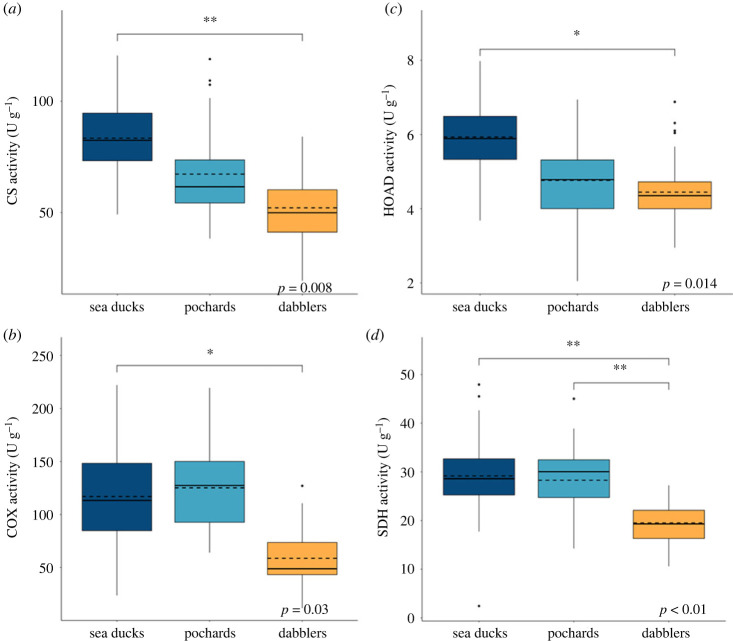
Maximal activity in the gastrocnemius for mitochondrial enzymes (*a*) citrate synthase (CS) and (*b*) cytochrome c oxidase (COX), and beta-oxidation enzyme (*c*) 3-hydroxyacyl-CoA dehydrogenase (HOAD), along with maximal activity in the pectoralis for mitochondrial enzyme (*d*) succinate dehydrogenase (SDH) for three tribes of ducks: sea ducks (diving, eight spp., *n* = 72), pochards (diving, three spp., *n* = 30), and dabblers (non-diving, five spp., *n* = 50). Activity was measured as μmol g^−1^ tissue min^−1^ (U g^−1^). Box plots show the median and quartile ranges of the data, with the mean indicated by the dashed line. In the gastrocnemius, values in the sea ducks are significantly higher than those in the dabblers, and in the pectoralis, values in both diving tribes are significantly higher than those in the dabblers using Bonferroni *post hoc* tests (*p < 0.05; **p < 0.01) after a phylogenetic ANOVA (for which *p*-values are shown on each panel).

**Figure 4.**
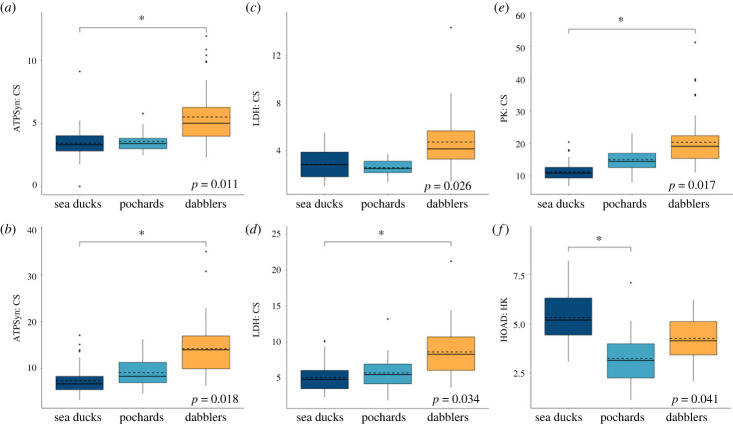
Enzyme activity ratios in three tribes of ducks: sea ducks (diving, eight spp., *n* = 72), pochards (diving, three spp., *n* = 30) and dabblers (non-diving, five spp., *n* = 50). Box plots show the median and quartile ranges of the data, with the mean indicated by the dashed line. There were significant decreases in the ratios in the sea ducks compared with the dabblers for ATPSyn:CS in both the (*a*) pectoralis and (*b*) gastrocnemius; in LDH:CS in the (*c*) pectoralis and (*d*) gastrocnemius; and in PK:CS in the (*e*) gastrocnemius. (*f*) HOAD:HK in the gastrocnemius is higher in the sea ducks than in the pochards. Bonferroni *post hoc* tests (*p < 0.05) were used after a phylogenetic ANOVA (for which *p*-values are shown on each panel).

**Figure 5.**
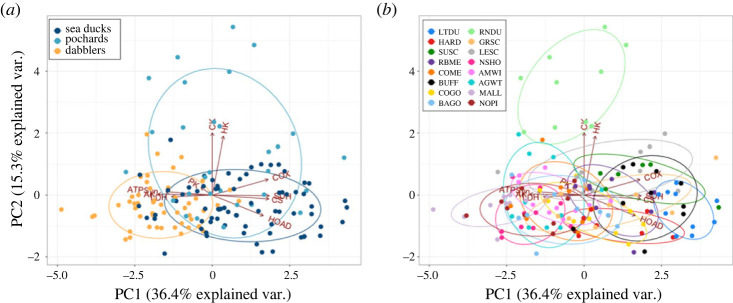
Principal components analysis (PCA) of the 10 enzymes assayed across all individuals in the gastrocnemius, coloured by (*a*) tribe, and (*b*) species. Ellipses represent the 95% confidence ellipse for each group. The three tribes are clearly visible along PC1, with the dabblers most distinct from the more deeply diverged sea ducks, and pochards intermediate between the two. Enzymes include hexokinase (HK); pyruvate kinase (PK); lactate dehydrogenase (LDH); hydroxyacyl-CoA dehydrogenase (HOAD); citrate synthase (CS); succinate dehydrogenase (SDH); cytochrome c oxidase (COX); ATP synthase (ATPSyn); creatine kinase (CK); adenylate kinase (AK). Species include long-tailed duck (LTDU); harlequin duck (HARD); surf scoter (SUSC); red-breasted merganser (RBME); common merganser (COME); bufflehead (BUFF); common goldeneye (COGO); barrow's goldeneye (BAGO); ring-neck duck (RNDU); greater scaup (GRSC); lesser scaup (LESC); northern shoveler (NSHO); American wigeon (AMWI); American green-wing teal (AGWT); mallard (MALL); northern pintail (NOPI).

The gastrocnemius appeared to have a significantly higher oxidative capacity in the sea ducks compared with the dabblers, with increased activity of CS (*p* = 0.008) and COX (*p* = 0.03) (figures 3 and 5), which may reflect a corresponding variation in mitochondrial abundance. However, there may have been a restructuring of oxidative phosphorylation in the sea ducks compared with the dabblers, as reflected by a reduced ATPSyn/CS ratio (*p* = 0.015; figure 4). Fuel oxidation capacities were also shifted in the gastrocnemius between sea ducks and pochards compared with dabblers. Both sea ducks and pochards showed a significant increase in HOAD activity (*p* = 0.014), a slight increase in the HOAD/HK ratio (*p* = 0.041), and decreases in the PK/CS (*p* = 0.01) and LDH/CS (*p* = 0.034) ratios, all of which could reflect an increased capacity for oxidizing lipids relative to carbohydrates. These differences were reflected in the PCA. Principle component 1 (PC1), which explained 36.4% of total variation, was the major axis separating the sea ducks and dabblers. On PC1, high positive loadings for CS, COX, SDH and HOAD separated the sea ducks from the negative loadings of PK, LDH, ATPSyn and AK that characterized the dabblers (figure 5). In the pochards, enzyme activity was generally intermediate between the sea ducks and dabblers, which is also visible in the PCA along PC1 (figure 5).

### (d) Pectoralis enzyme activity

Similar to the gastrocnemius, sea ducks and pochards exhibited decreased LDH/CS activity ratios in the pectoralis (*p* = 0.026; figure 4 and table 2), which may reflect a consistent decrease in the relative capacity for anaerobic metabolism across muscles in these two diving groups relative to dabblers. There may have also been a restructuring of oxidative phosphorylation in the pectoralis of both the sea ducks and pochards, as reflected by increased SDH activity (*p* = 0.001; figure 3 and table 1) and decreased ATPSyn/CS ratio (*p* = 0.011; figure 4 and table 2).

**Table 1.** Maximal activities of 10 metabolic enzymes in the pectoralis and gastrocnemius of 16 duck species, grouped by tribe: sea ducks (diving, eight spp., *n* = 72), pochards (diving, three spp., *n* = 30) and dabblers (non-diving, five spp., *n* = 50). All activities are in μmol g^−1^ tissue min^−1^ (U g^−1^). Hexokinase (HK); pyruvate kinase (PK); lactate dehydrogenase (LDH); hydroxyacyl-CoA dehydrogenase (HOAD); citrate synthase (CS); succinate dehydrogenase (SDH); cytochrome c oxidase (COX); ATP synthase (ATPSyn); creatine kinase (CK); adenylate kinase (AK). Data are shown as mean ± s.e.m. †Significantly different from dabblers in phylogenetic ANOVA Bonferroni *post hoc* tests (*p* < 0.05).

	pectoralis			gastrocnemius		
	sea ducks	pochards	dabblers	sea ducks	pochards	dabblers
*glycolysis and lactate production*						
HK	0.39 ± 0.02	0.29 ± 0.02	0.36 ± 0.02	1.16 ± 0.03	1.61 ± 0.09	1.09 ± 0.04
PK	1053.36 ± 15.65	1022.13 ± 30.80	1066.54 ± 16.71	907.34 ± 15.44	946.84 ± 26.10	967.77 ± 18.05
LDH	466.35 ± 20.19	391.99 ± 19.37	576.31 ± 23.39	399.31 ± 10.36	354.90 ± 17.41	411.21 ± 12.27
*beta-oxidation*						
HOAD	8.78 ± 0.08	8.55 ± 0.20	8.33 ± 0.18	5.93 ± 0.09 †	4.76 ± 0.20	4.45 ± 0.11
*citric acid cycle*						
CS	168.23 ± 3.53	153.85 ± 4.51	134.32 ± 4.92	83.41 ± 1.92 †	67.28 ± 3.68	52.13 ± 2.09
*electron transport system*						
SDH	29.17 ± 0.80 †	28.28 ± 1.33 †	19.49 ± 0.59	15.55 ± 0.64	12.38 ± 0.78	9.30 ± 0.52
COX	289.30 ± 19.77	382.10 ± 22.08	178.96 ± 13.35	117.00 ± 5.04 †	125.14 ± 7.85	58.69 ± 3.51
ATPSyn	566.44 ± 14.93	546.21 ± 24.40	673.64 ± 16.16	582.00 ± 16.66	567.19 ± 22.68	671.97 ± 18.36
*substrate-level phosphorylation*						
CK	15.43 ± 0.91	15.59 ± 1.11	24.74 ± 0.78	35.38 ± 1.19	44.19 ± 3.25	34.06 ± 0.86
AK	972.54 ± 22.12	847.98 ± 25.05	1035.00 ± 24.81	952.74 ± 29.94	928.44 ± 40.79	1127.81 ± 29.72

**Table 2.** Enzyme activity ratios in the pectoralis (P) and gastrocnemius (G) for 16 species of diving and non-diving ducks, grouped by tribe: sea ducks (diving, eight spp., *n* = 72), pochards (diving, three spp., *n* = 30) and dabblers (non-diving, five spp., *n* = 50). Hexokinase (HK); lactate dehydrogenase (LDH); hydroxyacyl-CoA dehydrogenase (HOAD); citrate synthase (CS); succinate dehydrogenase (SDH); cytochrome c oxidase (COX); ATP synthase (ATPSyn). Data are shown as mean ± s.e.m. †Significantly different from dabblers in phylogenetic ANOVA Bonferroni *post hoc* tests; *significantly different from pochards in phylogenetic ANOVA Bonferroni *post hoc* tests (*p* < 0.05).

muscle	tribe	CS:HOAD	LDH:CS	PK:CS	PK:LDH	SDH:CS	COX:CS	ATPSyn:CS	HOAD:HK
P	sea ducks	19.20 ± 0.38	2.85 ± 0.13	6.48 ± 0.18	2.57 ± 0.12	0.179 ± 0.006	1.76 ± 0.13	3.42 ± 0.13 †	29.71 ± 1.98
	pochards	18.17 ± 0.59	2.57 ± 0.11	6.75 ± 0.21	2.73 ± 0.11	0.187 ± 0.010	2.52 ± 0.16	3.57 ± 0.14	36.69 ± 3.61
	dabblers	16.17 ± 0.52	4.73 ± 0.33	8.67 ± 0.48	1.99 ± 0.08	0.154 ± 0.008	1.52 ± 0.19	5.51 ± 0.31	30.13 ± 3.24
G	sea ducks	14.23 ± 0.36	5.07 ± 0.22†	11.28 ± 0.32†	2.38 ± 0.07	0.188 ± 0.006	1.42 ± 0.06	7.37 ± 0.33†	5.29 ± 0.14*
	pochards	14.33 ± 0.61	5.73 ± 0.43	15.04 ± 0.76	2.86 ± 0.16	0.190 ± 0.012	1.90 ± 0.10	9.08 ± 0.57	3.24 ± 0.24
	dabblers	11.86 ± 0.46	8.65 ± 0.48	20.38 ± 1.13	2.46 ± 0.10	0.186 ± 0.011	1.15 ± 0.07	14.21 ± 0.81	4.24 ± 0.14

### (e) Phylogenetic generalized least-squares analysis of dive time

Select enzyme activities in the pectoralis or gastrocnemius, O_2_ carrying capacity ([Hb], [Mb]), and body mass were used as explanatory variables against dive time in phylogenetic least squares regressions (see Methods). In preliminary analyses, [Hb] and Hct were shown to be strongly collinear (*r*^2^ = 0.505, *p* < 0.001), so only [Hb] was retained in our models as an indicator of blood O_2_ carrying capacity. The best-fitting model (lowest AIC) to estimate dive time included all variables of interest: [Hb], [Mb] in the gastrocnemius, mass, the three enzymes in the gastrocnemius (HOAD, CS, COX) or pectoralis (SDH) that were significantly different between the sea ducks and pochards versus dabblers, and the two almost significantly different enzymes (SDH, HK, *p* < 0.10) in the gastrocnemius (electronic supplementary material, table S3). In this model, none of the variables was a significant predictor of dive time. Removal of SDH activity in the pectoralis to focus only on changes in the gastrocnemius only decreased the AIC by 0.3 (104.16 versus 104.46). When only gastrocnemius enzyme activity was considered, the increase in oxidative capacity seen in the sea ducks and pochards, as indicated by increased CS activity, was found to be the most significant predictor of dive time overall (*p* = 0.0379).

## 4. Discussion

Our results show significant remodelling of metabolic pathways in the locomotory muscle of the sea ducks, with a generally intermediate phenotype seen in the pochards. CS and COX are known to be biochemical indicators of mitochondrial abundance, and CS activity in the gastrocnemius was the best predictor of dive time [58–60]. Because CS and COX activities were increased in the sea ducks, an increase in the overall capacity for oxidative phosphorylation may be critical for increasing dive performance. HOAD was also elevated in the longer diving sea ducks, which could suggest a relative shift in the capacity for oxidizing lipids over carbohydrates.

### (a) Mitochondrial oxidative capacity

COX and CS activities were increased in the gastrocnemius of sea ducks, and CS activity in the gastrocnemius was the best predictor of dive time. As primarily leg-propelled divers, the increases in CS and COX in the gastrocnemius (the primary diving muscle) probably reflect an increased mitochondrial abundance, which is probably important for maximizing aerobic ATP supply to the muscle during locomotion. A similar trend is seen in pinnipeds, with increases in CS activity seen in swimming versus non-swimming muscles [10], and in penguin locomotory muscles as they transition from the nest to begin foraging at sea for themselves [11]. These findings suggest that mitochondrial O_2_ supply remains critical to maintaining aerobic metabolism while diving. Indeed, in the closely related tufted duck (*Aythya fuligula*), blood flow to the leg muscles was five times higher during a dive compared with resting levels, which probably helps meet the high demands for transporting circulatory O_2_ stores to the working muscle [61].

Myoglobin is important in muscles as both an intracellular O_2_ store and a facilitator of intracellular O_2_ diffusion. Work on both the tufted duck and marine mammals has shown that myoglobin content ([Mb]) is generally higher in swimming versus non-swimming muscles [5,62,63]. [Mb] is also higher in the sea ducks compared with the dabblers [46], which would increase muscular O_2_ availability during the dive, further extending aerobic dive times. Since we did not see consistent changes in the activities of either CK or AK, increased capacities for cytoplasmic ATP buffering and substrate-level phosphorylation do not appear to contribute to increases in dive capacity.

The increases in both CS and COX activity were not matched by an equivalent increase in ATP synthase activity, as evidenced by the decrease in the ATPSyn:CS ratio in the sea ducks and pochards. Interestingly, this trend was seen in both the gastrocnemius and pectoralis and could represent a specific diving phenotype in the muscle generally. However, ATP synthase activity remained relatively high across all three clades, and differences seen in the ATPSyn:CS ratio reflect the higher CS activity in the sea ducks and pochards. As a key enzyme involved in the first step of the TCA cycle, which provides energy-rich NADH and FADH_2_ to the electron transport chain, this increase in CS could function to maximize electrons funnelled into oxidative phosphorylation and creation of the protonmotive force for ATP synthesis. The lower ratio of ATP synthase to CS could also be indicative of an increase in the proportion of subsarcolemmal mitochondria, the subfraction of mitochondria located next to the cell membrane, which have a lower overall activity of ATP synthase compared with intermyofibrillar mitochondria [64]. Because subsarcolemmal mitochondria are adjacent to capillaries, their preferential enrichment may be useful for taking advantage of blood O_2_ stores during diving.

### (b) Capacities for oxidizing different metabolic fuels

Sea ducks and pochards had significantly elevated HOAD activity in the gastrocnemius in comparison with dabblers, probably reflecting a higher capacity for fuelling aerobic metabolism with fatty acids. Both sea ducks and pochards prey mostly on molluscs, crustaceans and fish, which generally have a higher lipid content than the vegetation preferred by the dabblers [65,66]. An increase in dietary lipids in birds is thought to upregulate the expression of aerobic enzymes, with positive correlations shown for both CS and HOAD [67,68]. Associated with the increase in HOAD activity in the sea ducks and pochards, we observed a small but significant increase in HOAD:HK and decreases in both PK:CS and LDH:CS, suggesting that the relative capacity for oxidizing carbohydrates is not similarly increased. Thus the mitochondria of diving birds may be specialized for lipid metabolism during breath-hold dives and/or between dive recovery.

If the increased capacity for fatty acid oxidation is reflected by increases in fatty acid use during dives, it could suggest that muscle metabolism is not restructured to maximally conserve O_2_. Fatty acid oxidation requires more O_2_ per mole ATP compared with carbohydrates, and a preference for carbohydrates is a strategy used by some taxa to improve exercise performance under low O_2_ conditions [55,69,70]. Mitochondrial fuel oxidation capacity differs between mitochondrial subfractions, with subsarcolemmal mitochondria having a greater relative capacity for oxidizing fats over carbohydrates compared with intermyofibrillar mitochondria [64,71,72]. Therefore, the differences in HOAD activity in diving ducks could also reflect an increased abundance of subsarcolemmal mitochondria in the muscle. Further investigations into mitochondrial distribution would confirm if this is in fact the case.

### (c) Metabolic changes in the long-tailed duck, the most extreme diver

Within the sea ducks, the long-tailed duck has the greatest dive time and dive depth [16], making it one of the strongest divers amongst species studied here. In addition, they were also one of the most distinct groupings along PC1 of the PCA (figure 5*b*). Compared with the other sea ducks, the long-tailed duck had the greatest CS and SDH activities and the lowest ATP synthase activity in the gastrocnemius (electronic supplementary material, table S4). The long-tailed duck was also an outlier for the activity of several enzymes in the pectoralis (electronic supplementary material, table S4). This suggests that the consistent remodeling of metabolic pathways that we observed in sea ducks is pushed to the extreme in the deepest diving duck among the group.

## 5. Conclusion

The locomotory muscles of sea ducks and pochards, but especially the sea ducks, appear to be specialized to sustain high rates of aerobic metabolism during diving locomotion. The combined increases in CS and COX activity probably reflect high overall capacities for oxidative phosphorylation, and high HOAD activities may enable high rates of lipid oxidation. Our findings also emphasize the importance of body O_2_ stores for extending dive capacity in diving ducks, because we found no evidence that diving ducks had consistently greater capacities for anaerobic metabolism or substrate-level phosphorylation. The present study highlights some of the key metabolic specializations that support increased dive performance across many species of specialized diving waterfowl.

## Data Availability

Raw enzyme data are available from the Dryad Digital Repository at: https://doi.org/10.5061/dryad.tht76hf4j [73]. Phylogeny data and code are provided in the supplementary material. ddRAD-Seq reads are available from NCBI's Sequence Read Archive BioProject PRJNA718623 (from [21]), and PRJNA1007384. Supplementary material is available online [74].
